# Translucent zirconia crown retention - effect of preparation, bonding and aging protocol

**DOI:** 10.1007/s00784-025-06447-6

**Published:** 2025-07-02

**Authors:** Nadja Rohr, Mark Rutscheidt, John Meinen, Marco Jäggi, Bogna Stawarczyk

**Affiliations:** 1 Biomaterials and Technology, Department Research, University Center for Dental Medicine Basel UZB, University of Basel, Mattenstrasse 40, Basel, 4058 Switzerland; 2 Department of Reconstructive Dentistry, University Center for Dental Medicine Basel UZB, University of Basel, Mattenstrasse 40, Basel, 4058 Switzerland; 3 Department of Prosthetic Dentistry, University Hospital, LMU Munich, Goethestraße 70, 80336 Munich, Germany

**Keywords:** Retention strength, Translucent zirconia, Resin composite luting material, Chewing simulation, Aging

## Abstract

**Objective:**

To obtain the retention strength of translucent zirconia crowns on human tooth abutments and evaluate effects of specimen preparation, bonding protocol, and comparing aging in two different chewing simulators.

**Materials and methods:**

Translucent zirconia crowns were bonded to prepared human teeth using two resin composite luting materials: Panavia V5 (PV5) and RelyX Universal (with primer [RUV] and without primer [RUO]). Specimens were fabricated at two sites (B and M) and the retention strength was determined before and after aging in two different chewing simulators (*N* = 216, *n* = 12) using 1.2 million cycles, 50 N, 1.5 Hz and 6000 thermal cycles. Data were analyzed with 3-way ANOVA and Bonferroni post-hoc test (α = 0.05).

**Results:**

Higher retention strength values were observed for specimens produced at site M (7.1 ± 2.6 MPa) compared to B (5.5 ± 2.7 MPa). Overall, highest values were obtained with PV5 (7.0 ± 2.1 MPa) and RUV (7.0 ± 2.7 MPa), while retention strength with RUO was lower (4.9 ± 2.9 MPa). The highest values overall were determined before aging (8.1 ± 1.8 MPa), followed by aging in the industrially available simulator using weight-loading (6.1 ± 2.4 MPa) and the custom-built hydraulic simulator (4.8 ± 2.9 MPa) enhancing aging effects.

**Conclusions:**

The specimen production affected retention strength values. Bonding protocols PV5 and RUV revealed similar results, while lower values were obtained using the self-adhesive approach RUO. Aging in the hydraulic simulator was more pronounced, despite identical settings.

**Clinical relevance:**

Differences in retention strength may occur between operators, despite strict bonding protocol adherence. An adhesive bonding protocol using the respective primer is recommended over self-adhesive bonding of translucent zirconia crowns on human teeth.

## Introduction

Zirconia is increasingly being used as the standard material for manufacturing single crowns (SCs) and fixed dental prostheses (FDPs). The survival rates for tooth-supported zirconia SCs and FDPs have become comparable to gold standard veneering ceramic fused to metal (PFM) restorations, with a 5-year survival summary estimate rate of 91.2% for SCs and 90.4% for multiple unit FDPs [1, 2]. The most common technical complication associated with restorations is reported to be chipping of the veneering ceramic, with an annual complication rate of 0.4% for SCs and 2.7% for FDPs [1, 2].

To minimize the risk of chipping, there is a growing trend towards the use of monolithic zirconia materials. New, translucent zirconia materials containing an increased amount of yttria (4–5 mol%) have been developed for this purpose [3, 4]. However, the flexural strength of these materials decreases compared with opaque framework zirconia materials from 1200 MPa to 600–800 MPa [5]. The latest materials even combine different amounts of yttria within the same blank, allowing for a gradient in translucency and strength [6–8]. Due to the lack of long-term clinical studies, the recommendation for clinical application of translucent zirconia materials is still limited [9].

Adhesive bonding of zirconia crowns is conducted using either an adhesive or self-adhesive resin composite luting material [10]. Aiming simplification of the bonding procedure, universal adhesives are available, allowing for the pre-treatment of both, the tooth substance, and the restoration [11, 12]. Even though, in-vitro studies with shear bond strength testing exist, the efficiency of such novel bonding approaches has not yet been simulated with actual tooth-supported translucent zirconia crowns in a chewing simulator [11, 13–16].

While other aging mechanisms, such as thermal cycling, are commonly used, simulating aging in a chewing simulator for 5 years with 1.2 million cycles and 50 N force application combined with thermal cycling is a frequently applied method [17–19]. However, the applied parameters (force, frequency, number of cycles, thermal aging temperature) often vary widely among research institution. Currently, only one company (SD Mechatronik) commercially distributes chewing simulators in various designs in Europe, while other institutions have custom-built models (e.g., Model Zurich). As the chewing simulators use different loading mechanisms, a direct comparison of the aging effect under the same experimental setup would be of high interest for the comparability of future studies. A comparison between different chewing simulators with the same set-up has not yet been done. Additionally, the specimen preparation by various operators can also have an effect on the outcome [20, 21].

This study aims to examine how the preparation of SC specimens, the choice of adhesive bonding protocol, and the type of chewing simulator impact the retention strength values of translucent zirconia crowns on human tooth abutments. The following null hypotheses will be tested: (1) The retention strength is not influenced by the specimen preparation, (2) the retention strength is not influenced by the adhesive bonding protocol, (3) the retention strength is not influenced by the type of chewing simulator.

## Materials and methods

**Fig. 1:**
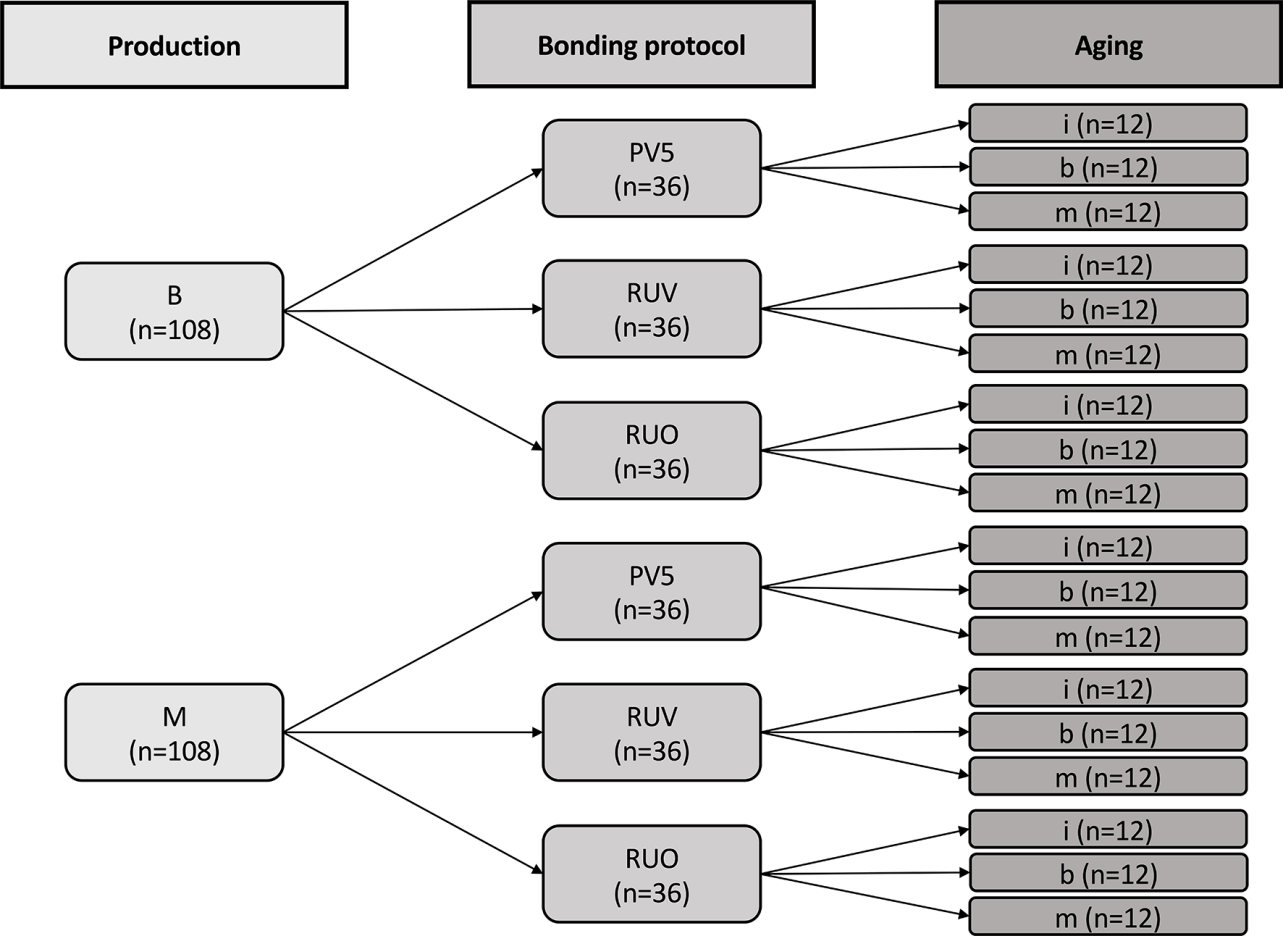
Test set-up. Specimens were either prepared at location B or M with the subsequent bonding protocols. Specimens were either not aged (i) or subjected to chewing simulation using a customized model (b) or an industrially available device (m). PV5: Panavia V5 with primers; RUV: RelyX with primer; RUO: RelyX without primer

The retention strength of 216 zirconia crowns on prepared human molar teeth was measured followed by fracture analysis. The specimen preparation was conducted at two different research institutions (B: Basel and M: Munich). The crowns were fabricated using a translucent multi-layered zirconia disk with a 5 mol% yttria partially stabilized zirconia (5Y-PSZ) enamel and 4Y-PSZ dentin layer (IPS e.max Prime Esthetic, Ivoclar, Schaan, Liechtenstein) and bonded to the prepared abutment teeth. Three different bonding protocols were applied using a clinically established adhesive (Panavia V5 (PV5), Kuraray Noritake, Okayama, Japan) and a self-adhesive resin composite luting material (RelyX Universal, 3M, Neuss, Germany) with (RUV) and without primer (RUO). Either no aging was applied, or specimens were submitted to chewing simulation using the same parameters at either research institution (b and m). The test set-up is displayed in Fig. 1 and materials are given in Table 1.

**Table 1:** Material compositions obtained from manufacturer’s information and safety data sheets

Type	Name	Manufacturer	Composition	LOT-Nr.
Luting material				
Adhesive resin composite luting material	Panavia V5	Kuraray Noritake, Okayama, Japan	Bis-GMA, TEGDMA, hydrophobic aromatic dimethacrylate, hydrophilic aliphatic dimethacrylate silanated barium glass filler, silanated fluoroalminosilicate glass filler, colloidal silica, silanated aluminum oxide filler, dl-camphorquinone, initiators, accelerators, pigments	BH0220
(Self-) adhesive resin composite luting material	RelyX Universal	3M, Neuss, Germany	HEMA, UDMA, TEGDMA, phosphorylated dimethacrylate adhesion monomers, ytterbium trifluoride, glass powder surface modified, silane, trimethoxyoctylhydrolysis products with silica, triphenyl phosphite, t-amil hydroperoxide, 26-ditert-butyl-P-cresol	9,648,612
**Primer**				
Ceramic primer	Clearfil Ceramic Primer Plus	Kuraray Noritake, Okayama, Japan	3-methacryloxypropyl trimethoxysilane, MDP, ethanol	AT0087
Tooth primer	Panavia V5 Tooth Primer	Kuraray Noritake, Okayama, Japan	MDP, HEMA, hydrophilic aliphatic dimethacrylate, accelerators, water	AR0122
Universal primer	Scotchbond Universal Adhesive Plus	3M, Neuss, Germany	Dimethacrylate monomers, MDP, HEMA, copolymer of acrylic and itaconic acid, (3-aminopropyl) triethoxysilane, silica filler, camphorquinone, N,dimethylbenzocaine, acetic acid, ethanol, water	B: 7,988,488 M: 9,505,283
**Zirconia**				
Zirconia	IPS e.max ZirCAD Prime Esthetic, 98.5 × 16 mm	Ivoclar, Schaan, Lichtenstein	> 86.00 wt% ZrO_2_, 6.50–8.30 wt% Y_2_O_3_, ≤ 5 wt% HfO_2_, 0.03–0.05 wt% Al_2_O_3_, < 1.00 wt% colorants and sintering additives	Z04 × 95

Abbreviations: Bis-GMA: bisphenol A diglycidyl methacrylate; HEMA: 2-hydroxyethyl-methacrylate; MDP: 10-methacryloyloxydecyl dihydrogen phosphate; TEGDMA: triethylene glycol dimethacrylate; UDMA: urethane dimethacrylate

### Specimen production

**Fig. 2:**
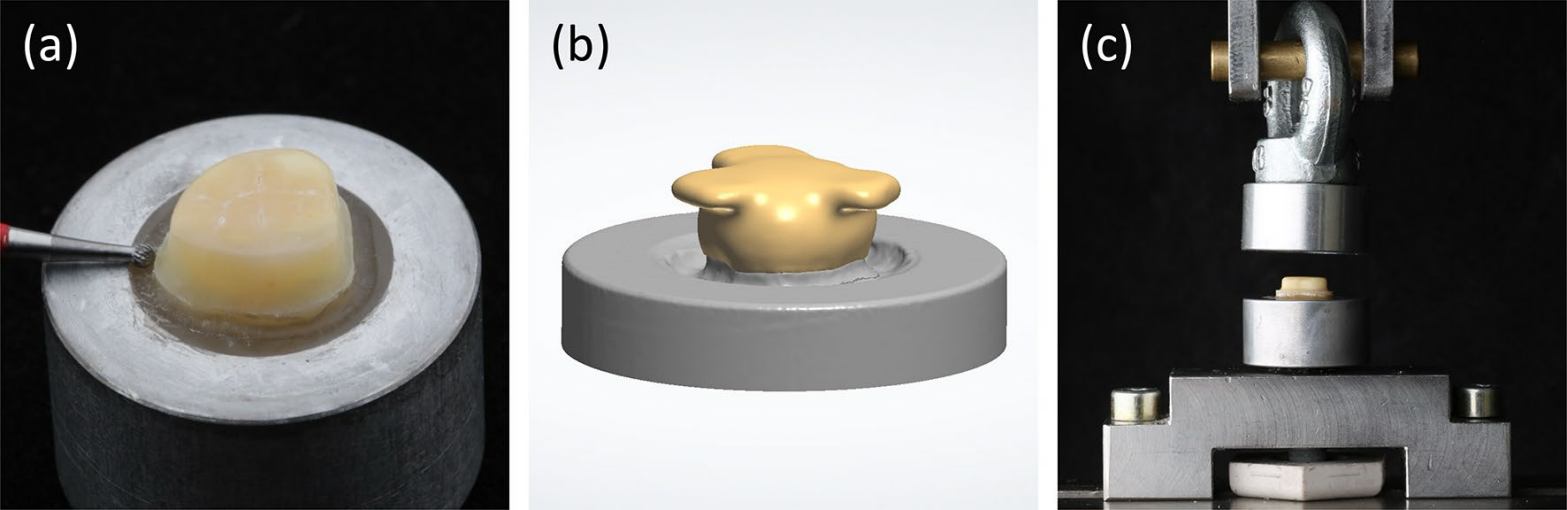
Test set-up: (**a**) Final prepared abutment (**b**) Designed zirconia crown for the abutment (**c**) Retention testing device attached to the universal testing machine

For this study 216 extracted caries-free human molars were included. The use of extracted human teeth was approved by the local ethic committee (EKNZ UBE- 15/111). The teeth were cleansed of debris and residual soft tissue structures and stored in 0.5% chloramine T (Carl Roth, Karlsruhe, Germany) at room temperature for the first 7 days. The teeth were then stored in distilled water at 5 °C for a maximum of 6 months (ISO 11405:2015). The teeth were embedded in cylindrical lower steel embedding molds with a dimension of (25 mm diameter outside, 15 mm inside top) using dental boxing wax (Kerr, Nobel Biocare Services, Kloten, Switzerland) and autopolymerizing acrylic resin (Scandiquick, Scan-Dia Materialographie, Hagen, Germany). The embedded teeth were then positioned on a custom-made device in a parallelometer (B: S3 Master, Schick GmbH, Schemmerhofen, Germany; M: F4 Basic, Degudent, Hanau, Germany) and reduced to a chamfered preparation with a conicity of 10° per side using a diamond rotary instrument (Torpedo 14 8879314014, Komet Dental, Lemgo, Germany; Perfecta 900, W & H Dentalwerk, Bürmoos, Austria) (Fig. 2a). The abutment height was subsequently reduced to 3 mm using a cut-off grinder (B: Accutom 100, Struers, Ballerup, Denmark/ M: Secotom 50, Struers). The coronal edges were rounded with polishing discs (3 M Sof-Lex, 3 M, Saint Paul, Minnesota, USA).

After preparation, each abutment was scanned individually (B: 3Shape E4, 3shape, Copenhagen, Denmark / M: Ceramill map400, Amann Girrbach, Koblach, Austria). The STL files were imported into a surveying software (GOM Inspect, Carl Zeiss GOM Metrology, Braunschweig, Germany) to calculate the surface area of the bonding interface. The crowns were designed (B: 3Shape Dental System, 3Shape / M: Ceramill Mind-ExoCAD V.4.6, Amann Girrbach) with a flat occlusal surface and three retentive elements (Fig. 2b). The layer thickness of the restoration was set to 1.5 mm and the luting material gap to 0.05 mm. The crowns were nested in the middle layer of the multi-layered zirconia disk (IPS e.max ZirCAD Prime Esthetic A3 16 mm, Ivoclar). This blank has a translucent 5Y-PSZ layer of 4 mm, translation layer of 3 mm and 4Y-PSZ dentin layer of 9 mm in height. Hence, specimens were placed in the transition zone and dentin material. Crowns were then milled dry (B: PM7, Ivoclar / M: PM7, Ivoclar) using the setting standard milling coated tools and sintered (B: Programat S2, Ivoclar / M: Nabertherm LHT02/16, Nabertherm, Lilienthal, Germany) according to the manufacturer’s instructions.

### Bonding protocol

The intaglio surfaces of the crowns were air-borne particle abraded with 50 μm Al₂O₃ (ORBIS Dental Handelsgesellschaft, Münster, Germany) for 10 s, angle: 45°, pressure: 0.1 MPa, distance: 10 mm and subsequently cleaned in an ultrasonic bath for 3 min with 70% ethanol. The prepared abutment teeth were stored in distilled water at 4 °C until use and cleaned with pumice before bonding. For the bonding procedure the teeth from sites B (*n* = 108) and M (*n* = 108) were each further divided into 3 subgroups.

Group **PV5**: (B: *n* = 36; M: *n* = 36) A ceramic primer (Clearfil Ceramic Primer plus, Kuraray Noritake) was applied for 20 s with a microbrush to the intaglio surface of the crowns followed by drying. The abutment surfaces were gently dried and treated with a self-etching Primer (Panavia V5 Tooth Primer, Kuraray Noritake) using a micro-brush and subsequent air-drying. The crowns were then bonded to the prepared teeth using an adhesive resin composite luting material (Panavia V5, Kuraray Noritake).

Group **RUV**: (B: *n* = 36; M: *n* = 36) A universal adhesive (Scotchbond Universal Plus Adhesive, 3M) was applied to the intaglio surface of the crown as well as on the gently dried abutment surface with a microbrush for 20 s, followed by air-drying. Crowns were bonded with (self)-adhesive resin composite luting material (RelyX Universal, 3M).

Group **RUO**: (B: *n* = 36; M: *n* = 36) application of (self)-adhesive resin composite luting material (RelyX Universal, 3M) without further pre-treatment of the gently dried abutments or air-abraded crowns.

The crowns were loaded with 20 N using a custom-built device for pressure application during bonding. Excess was removed using a micro-brush. Subsequently, all specimens were light cured from 2 opposing sides and from occlusal for 20 s each (Elipar DeepCure-S, 1,200 mW/cm^2^, 3M). All specimens were stored in distilled water at 37 °C for 24 h to allow further polymerization and kept in distilled water at room temperature for another 6 days due to transportation time between the two research institutions.

### Aging

For the non-aged group (i) the retention strength was obtained 7 days after production (*n* = 72) due to transportation to site M, while the remaining two test groups (b: *n* = 72 and m: *n* = 72) were exposed to aging in two different chewing simulators. The chewing simulation was performed using custom-built model Zurich (b), which is based on a hydraulic system whereby each of the 12 chambers is controlled individually. The industrially available chewing simulator (m) (CS 4.8, SD Mechatronik, Feldkirchen-Westerham, Germany) loads all 8 chambers simultaneously by a weight of 5 kg lowered on the specimens. For both chewing simulators, the parameters were set as follows: 1.2 million cycles, 50 N, 1.5 Hz, 6000 thermal cycles 5 °C / 55 °C. Steatite balls simulating a standardized elastic modulus of human enamel with a diameter of 6 mm (SD Mechatronik) served as antagonists and specimens were loaded in the center in axial direction. After completing aging, all specimens were again stored in water at room temperature for 7 days due to transportation to site M for retention strength testing.

### Retention strength and failure analysis

To obtain the retention force, the bonded crowns were embedded in a second cylindrical mold. To ensure that both retaining devices were aligned parallel, a 1.5 mm layer of C-silicone (ORBIS Dental Handelsgesellschaft, Münster, Germany) was applied in the space between them. Next an autopolymerizing acrylic resin (Scandiquick, Scan-Dia Materialographie) was poured through the screw hole at the bottom of the upper holding device. Subsequently, the specimens were fixed in a custom-built device for pull-off tests in a universal testing machine (RetroLine, Zwick/Roell, Ulm, Germany) (Fig. 2c). A crosshead speed of 0.5 mm/min was applied until failure occurred and the maximum load was recorded. The retention strength value was calculated for each specimen individually using the following formula: Maximum load / bonding area = N/mm^2^ = MPa.

**Fig. 3:**
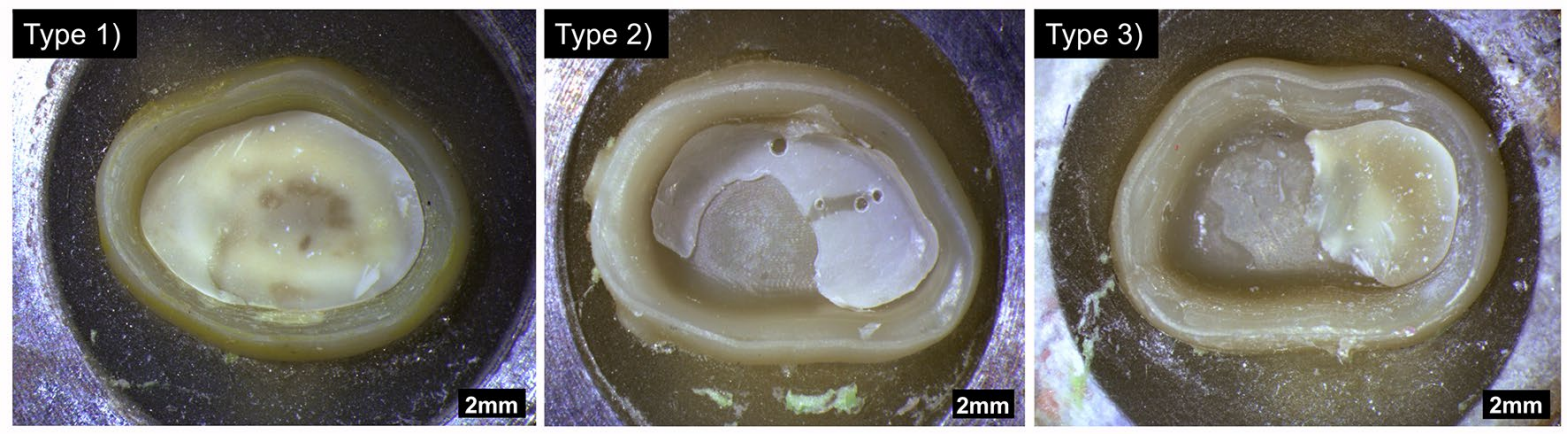
Light microscope images of the failure types displaying the intaglio surfaces of the debonded crowns. Type (1) cohesive failure; Type (2) adhesive failure; Type (3) cohesive/adhesive mixed failure

The failure types after testing were divided into three main groups (Fig. 3):

1) cohesive failure within the tooth structure.

2) adhesive failure, resin composite luting material remains in crown, on the abutment or on both.

3) cohesive/adhesive mixed failure. A part of the tooth abutment is still attached to the crown, while in another part, there has been an adhesive failure.

The debonding surface was examined by two operators using a light microscope (Wild M7A, Wild, Heerbrugg, Switzerland) with 12x magnification.

### Statistical analysis

The number of specimens per group was determined by a power analysis using data from a previously conducted study with 10 specimens per group (power 91.7%) [22]. The number was increased to 12 to account for the influence of 3 different factors (specimen production, bonding protocol, aging). The retention strength data were checked for normal distribution with Shapiro-Wilk-test. A 3-way ANOVA was applied to evaluate the effects of specimen production, bonding protocol, and aging. Differences between sub-groups were evaluated with Bonferroni post-hoc test (α = 0.05).

## Results

**Fig. 4:**
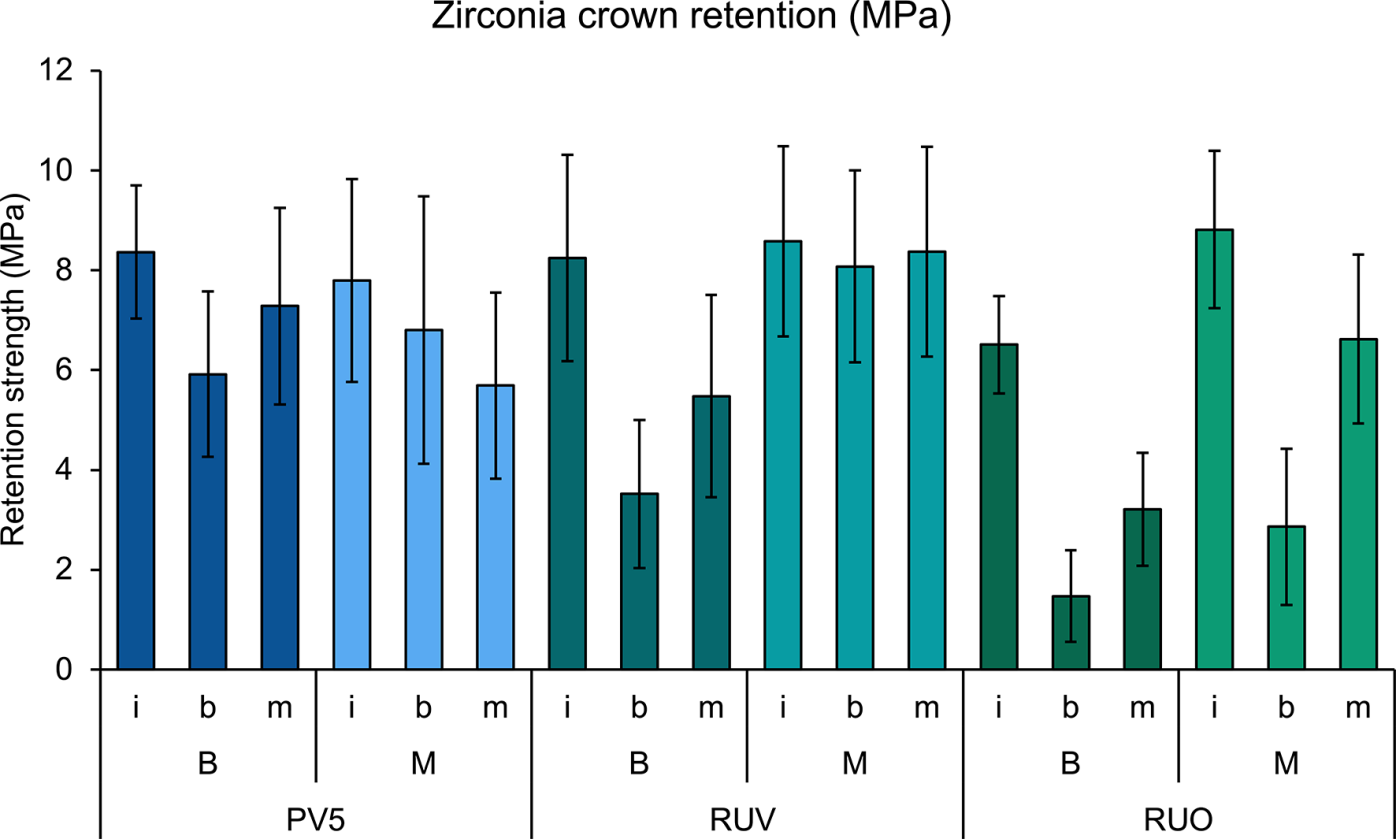
Retention strength mean and standard deviation of translucent zirconia crowns on human teeth. Crowns were prepared at B or M and bonded using either PV5, RUV or RUO bonding protocol. Measurements were conducted without aging (i) or after aging in the custom-built chewing simulator (b) or industrially available chewing simulator (m)

**Fig. 5:**
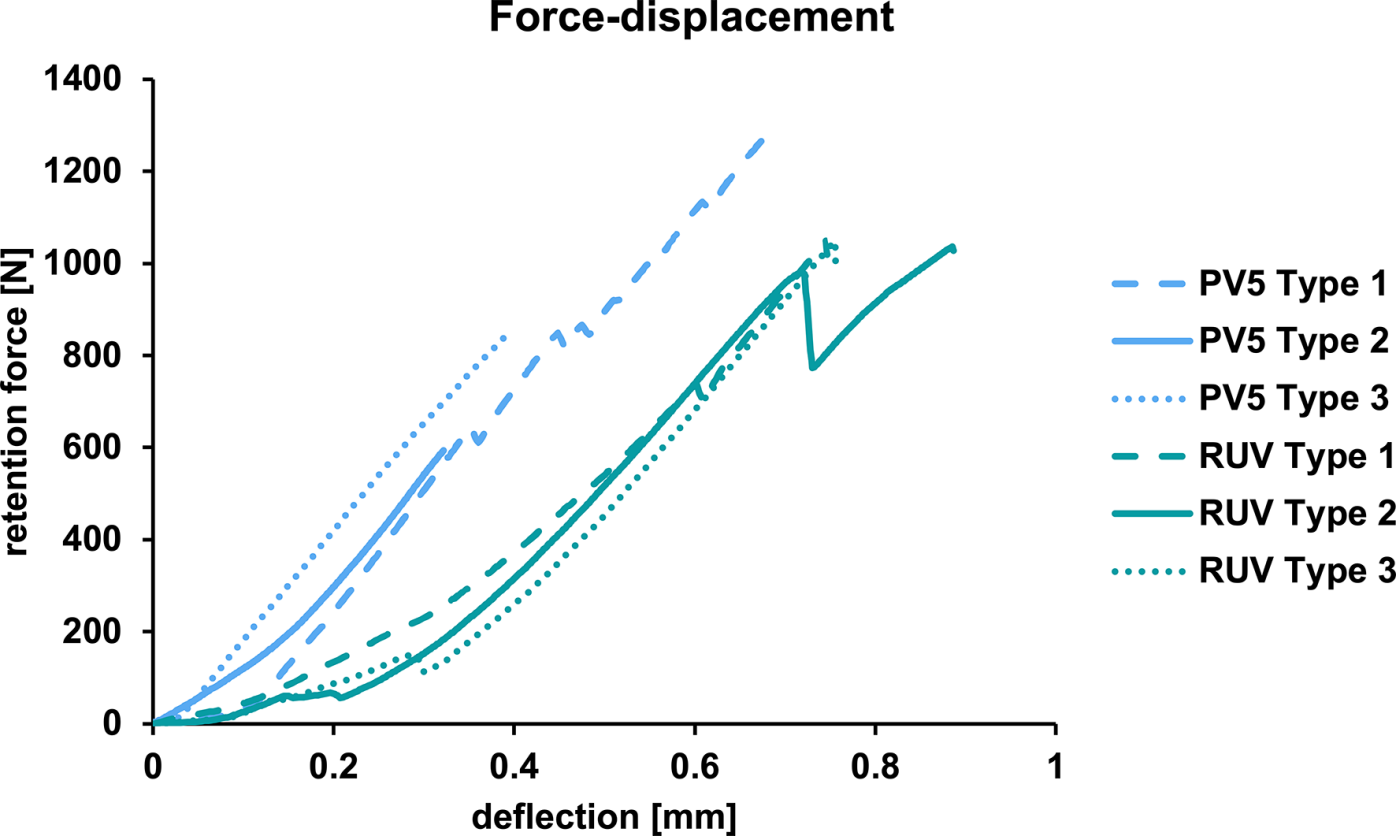
Force-displacement of one specimen per failure type that occurred in groups M-PV5-b and M-RUV-m as an example during retention testing

All specimens survived aging in the chewing simulator without visible damage or debonding. The retention strength mean values with standard deviation for each group are presented in Fig. 4; Table 2. An example for force-displacement during retention strength testing is given in Fig. 5 (RUO-B-I). After retention testing, 3 specimens were excluded from the analysis due to embedding issues of the crown within the second holder. The mean retention strength value varied between 1.5 ± 0.9 MPa (RUO-B-b) and 8.8 ± 1.6 MPa (RUO-M-i). All individual groups except RUV-B-i and RUO-B-b were normal distributed. Three-way ANOVA demonstrated an effect of specimen production, bonding protocol and aging on retention strength values (all *p* < 0.001) (Table 3).

Regarding specimen fabrication, overall, higher values were observed for specimens produced at site M (7.1 ± 2.6 MPa) compared with those at B (5.5 ± 2.7 MPa) (*p* < 0.001). For the bonding protocol the highest values overall were obtained for PV5 (7.0 ± 2.1 MPa) and RUV (7.0 ± 2.7 MPa) with no difference between them (*p* = 1.000). Both were higher than RUO that was applied in self-adhesive mode (4.9 ± 2.9 MPa) (*p* < 0.001). For factor aging, non-aged specimens of group i exhibited the highest values (8.1 ± 1.8 MPa), followed by aging in the industrially available chewing simulator type m (6.1 ± 2.4 MPa) and the customized model type b (4.8 ± 2.9 MPa) (all *p* < 0.001).

**Table 2:** Retention strength values (mean and SD) in mpa. Retention force values in Newton (N). Values of the prepared bonding area of the abutments (mean and SD) in mm^2^. Results of the fracture types (Type 1: cohesive failure, type 2: adhesive failure, type 3: cohesive/adhesive mixed failure) in %

Bonding protocol	Production	Aging	Retention strength (MPa)	Retention force (*N*)	Area (mm^2^)	Fracture type (%)		
**PV5**	**B**	**i**	8.4 ± 1.3	834 ± 147	99.8 ± 10.5	1) 83.3	2) 16.6	3) 0.0
		**b**	5.9 ± 1.7	721 ± 217	120.1 ± 11.5	1) 72.9	2) 18.2	3) 9.1
		**m**	7.3 ± 2.0	831 ± 290	112.3 ± 16.7	1) 91.7	2) 8.3	3) 0.0
	**M**	**i**	7.8 ± 2.0	1166 ± 358	149.0 ± 19.4	1) 81.9	2) 0.0	3) 18.2
		**b**	6.8 ± 2.7	968 ± 427	141.7 ± 14.9	1) 66.6	2) 0.0	3) 33.3
		**m**	5.7 ± 1.9	657 ± 210	117.0 ± 18.9	1) 25.0	2) 50.0	3) 25.0
**RUV**	**B**	**i**	8.2 ± 2.1	915 ± 244	111.2 ± 14.9	1) 91.7	2) 0.0	3) 8.3
		**b**	3.5 ± 1.5	403 ± 172	114.9 ± 13.7	1) 8.8	2) 91.6	3) 0.0
		**m**	5.5 ± 2.0	670 ± 241	122.8 ± 10.3	1) 41.6	2) 50.0	3) 8.3
	**M**	**i**	8.6 ± 1.9	1023 ± 192	121.5 ± 19.6	1) 91.7	2) 0.0	3) 8.3
		**b**	8.1 ± 1.9	978 ± 285	120.1 ± 13.2	1) 91.7	2) 8.3	3) 0.0
		**m**	8.4 ± 2.1	1124 ± 302	135.2 ± 23.5	1) 58.4	2) 8.3	3) 33.3
**RUO**	**B**	**i**	6.5 ± 1.0	721 ± 183	109.8 ± 17.2	1) 66.6	2) 25.0	3) 8.3
		**b**	1.5 ± 0.9	171 ± 96	119.8 ± 13.1	1) 0.0	2) 83.3	3) 16.7
		**m**	3.2 ± 1.1	338 ± 157	101.8 ± 13.6	1) 0.0	2) 100.0	3) 0.0
	**M**	**i**	8.8 ± 1.6	1017 ± 287	115.1 ± 24.2	1) 58.3	2) 25.0	3) 16.7
		**b**	2.9 ± 1.6	317 ± 169	112.3 ± 13.8	1) 0.0	2) 100.0	3) 0.0
		**m**	6.6 ± 1.7	809 ± 318	116.4 ± 21.7	1) 18.2	2) 54.5	3) 27.3

**Table 3:** Three-way ANOVA table of retention strength values (*p* < 0.05)

Factors	SS	d.f.	MS	F	*p*-level	Omega Sqr.
Production	126.3	1	126.3	40.6	< 0.001	0.08
Resin	218.7	2	109.3	35.2	< 0.001	0.13
Aging	399.6	2	199.8	64.3	< 0.001	0.24
Production x Resin	96.0	2	48.0	15.4	< 0.001	0.06
Production x Aging	26.5	2	13.3	4.3	0.015	0.01
Resin x Aging	93.7	4	23.4	7.5	< 0.001	0.05
Production x Resin x Aging	58.0	4	14.5	4.7	< 0.001	0.03
Within groups	606.3	195	3.1			
Total	1625.1	212	7.7			
Omega squared for combined effect	0.6					

### Failure type

Failure types occurring in % within all examined groups are given in Table 2. In general, it was observed that fracture types aligned congruently with the mean values of the bond strength test. Meaning higher retention strength values were more likely to result in cohesive failures within the tooth structure and lower values correlated with adhesive fractures within the bonding interface. The mean value for a fracture of type (1) was 8.0 MPa, for type (2) 3.9 MPa and for type (3) 6.8 MPa. In groups i where no aging was applied, cohesive fractures were predominant. Additionally, cohesive fractures were predominant in the PV5 Group after aging, except for PV5-M-m. Similarly, the RUV-M group exhibited cohesive fractures after both aging types. Adhesive fractures were mainly observed after the aging process in both RUO groups and in the RUV-B group. The force-displacement curve for PV5 inclined at a lower deflection than RUV, irrespective of the failure mode (Fig. 5).

## Discussion

This study revealed that the 3 factors specimen production, bonding protocol as well as the type of chewing simulator influenced retention strength of zirconia single crowns on human teeth, rejecting all three null hypotheses. To simulate the survival of the specimen in the laboratory, the crown removal test was chosen to evaluate the retention strength between the zirconia crown and the tooth abutment. Although there is no consensus on the best method for testing zirconia-resin or luting material-tooth interfaces, shear bond strength tests between two substrates are often conducted as screening tests for different surface treatments due to the more cost-efficient specimen preparation [23, 24]. Obtaining the retention strength of crowns provides a more clinically relatable outcome, however, as observed, specimen preparation and the chosen aging method can impact the outcome, hence, the obtained retention strength values are only comparable within the same study set-up. Additionally, for interpretation it must be considered that the calculated retention strength values of cohesively fractured specimens of failure type 1 and 3 are based on the bonded and not the fractured area. In these cases, the adhesive bond was stronger than the intrinsic strength of the tooth substrates. Despite these limitations, this study provides relevant findings for the clinical use of adhesive bonding protocols and identifies factors contributing to bonding failure.

For this study, human teeth were used which were stored according to the ISO 11,405 after extraction. The teeth were collected at the same location (B). However, as organic human material was used, individual structural differences occurred. For future studies it would be advisable to choose a similar range of tooth sizes within each group as the mean bonding area varied between the test groups. Even though the preparation procedure was standardized as detailed as possible, small differences appear to have contributed to an effect of the factor specimen production on retention strength values. The teeth were prepared manually in a parallelometer by the same operators in B (MR) and M (JM), yet subtle variations in hand pressure, water cooling, or instrument guidance could not be entirely excluded. Additionally, due to anatomical differences such as varying fissure depth, small amounts of enamel may have remained in the occlusal area, resulting in slight differences in the amount of enamel removal. However, such variability also mirrors clinical conditions: enamel thickness can differ substantially between patients depending on age, dietary habits, or geographical background. From a practical standpoint, adhesive luting systems must achieve reliable performance despite these individual anatomical differences and varying preparation geometries. Therefore, the inclusion of such natural variability may, in fact, enhance the clinical relevance of the present study. No further tests were carried out, but by visual comparison of STL files of the prepared abutments, it can be assumed that M used a more invasive preparation in the occlusal area and therefore eliminated the enamel completely. While for B, small enamel spots in the occlusal fissure area may have been present for some prepared specimens. In a previous study where the shear bond strength of RelyX Universal was measured to human teeth, shear bond strength was higher to dentin than enamel, with and without prior Primer application while no differences were observed with Panavia V5 [11]. This would explain the higher values for the groups bonded with RelyX Universal prepared in M as potentially more dentin area was available for bonding. These preparation differences would also explain why the PV5 group produced in B had comparable retention strength values as those in M. Another study showed that the bond strength of RelyX Universal decreased compared to Panavia V5 when bonded to dentin after thermal aging, whereas no difference was found for Panavia V5 [25]. These findings were confirmed in this study, where PV5 was less affected by aging. According to the manufacturer, RelyX Universal can be used as in self-adhesive or adhesive mode in combination with the respective primer. This study showed that adding the primer resulted in overall higher retention strength values.

To age the specimens and simulate intraoral conditions as closely as possible, they were subjected to 1.2 million cycles at 50 N, 1.5 Hz, with 6000 thermal cycles between 5 °C and 55 °C which is reported to simulate intraoral aging of 5 years [19]. To simulate intraoral conditions as closely as possible, it would be best to choose human enamel as antagonist [26]. Steatite beads were used in the study, which have no measurable differences in the effect of wear on zirconia compared to enamel and provide standardized geometrical conditions [27, 28]. No study has yet directly compared two chewing simulators of different design with the same parameters (1.2 million cycles, 50 N, 1.5 Hz, 6000 thermal cycles 5 °C/55°C). This study shows a lower retention strength values after aging using the custom-built chewing simulator (b) compared with the commercially available simulator (m). The custom-built simulator is based on a hydraulic-pneumatic system in which each of the 12 chambers can be individually controlled and the force is adjusted individually. However, it is not possible to change the horizontal position of the specimens in the chewing simulator due to the fixed holding device. This means that the teeth must be embedded exactly centered in the cylindrical molds. In contrast, the chewing simulator in M (CS 4.8, SD Mechatronik) loads all 8 chambers simultaneously with one weight and has no individual adjustment during testing. But it is possible to align the already embedded abutment so that the antagonist is centered on the crown.

When analyzing failure modes, it was observed that adequate bonding of the crowns to the tooth abutment was achieved, due to the frequent occurrence of cohesive fracture, hence the limiting factor was the strength of the tooth structure and not the interface between tooth and crown [24]. The long-term bond strength of zirconia was previously considered critical due to the limited number of test groups evaluated under aged conditions [29]. This study revealed that, when using adequate adhesive bonding protocols, such as air-borne-particle abrasion of translucent zirconia followed by application of a primer containing 10-methacryloyloxydecyl dihydrogen phosphate (MDP) a retention strength higher than the strength of the tooth structure can be expected. The retention strength of translucent zirconia crowns can be enhanced after simulated aging by choosing an adhesive over a self-adhesive bonding protocol.

## Conclusion

The hypotheses investigated in this study - whether the specimen preparation, the bonding protocol and the type of chewing simulator influence the retention strength of zirconia crowns on human tooth abutments were rejected. Within the limitations of this study, the following conclusions were drawn:

1. The specimen preparation had an influence on retention strength of translucent zirconia crowns, especially with RelyX Universal.
2. The different resin composite luting materials showed a difference in retention strength. Panavia V5 and RelyX Universal in combination with the respective primers displayed highest retention strength. The self-adhesive approach for RelyX Universal without primer application showed reduced retention strength.
3. Chewing simulation decreased retention strength values. When comparing the two chewing simulators, aging in the custom-built model was more efficient, hence retention strength values were lower than when using the industrially available model, highlighting that even when using identical settings for chewing simulators, outcomes should not be directly compared. Further measures are therefore necessary to standardize simulated aging protocols and devices in dentistry to allow for future comparability of studies.

## Data Availability

The data supporting the presented results can be viewed upon a reasonable request to the corresponding author.

## Abbreviations

B: Specimen production in Basel
M: Specimen production in Munich
i: initial, without aging
b: Aging in custom-built chewing simulator in Basel
m: Aging in industrially available chewing simulator in Munich
PV5: Panavia V5 in combination with Clearfil Ceramic Primer Plus and Panavia V5 Tooth Primer
RUV: RelyX Universal in combination with Scotchbond Universal Adhesive
RUO: RelyX Universal without Scotchbond Universal Adhesive
